# Exploring the Feasibility, Acceptability, and Safety of a 4:3 Intermittent Fasting Weight Loss Program Among Stage I-III Breast Cancer Survivors With Overweight or Obesity: Protocol for a 6-Month Proof-of-Concept Feasibility Trial

**DOI:** 10.2196/86281

**Published:** 2026-07-23

**Authors:** Katrina M Oselinsky, Emily B Hill, Danielle M Ostendorf, Zhaoxing Pan, Kristen Bing, Liza T Wayland, Chloe Simpson, Kevin C Clark, Anaeli Sandoval, Elizabeth A Thomas, Ryan J Marker, Peter Kabos, Paul S MacLean, Victoria A Catenacci

**Affiliations:** 1 Division of Endocrinology, Metabolism, and Diabetes, Department of Medicine, University of Colorado Anschutz Medical Campus, 12348 E Montview Blvd, Aurora, CO, 80045, United States, 1 303-724-0624; 2 Anschutz Health and Wellness Center, University of Colorado Anschutz Medical Campus, Aurora, CO, United States; 3 University of Colorado Cancer Center, Aurora, CO, United States; 4 Section of Nutrition, Department of Pediatrics, University of Colorado Anschutz Medical Campus, Aurora, CO, United States; 5 Department of Kinesiology, Recreation, and Sport Studies, University of Tennessee at Knoxville, Knoxville, TN, United States; 6 Section of Endocrinology, Department of Pediatrics, University of Colorado Anschutz Medical Campus, Aurora, CO, United States; 7 Division of Endocrinology, Rocky Mountain Regional VA Medical Center, Aurora, CO, United States; 8 Research Service, Rocky Mountain Regional VA Medical Center, Aurora, CO, United States; 9 Department of Physical Medicine and Rehabilitation, University of Colorado Anschutz Medical Campus, Aurora, CO, United States; 10 Division of Medical Oncology, Department of Medicine, University of Colorado Anschutz Medical Campus, Aurora, CO, United States

**Keywords:** cancer, diet, physical activity, intermittent fasting, behavioral weight loss

## Abstract

**Background:**

Mounting evidence underscores the adverse effects of excess body weight during cancer survivorship, yet many breast cancer survivors struggle to maintain a healthy weight (approximately 70% have overweight or obesity [OW/OB]). Daily caloric restriction with increased physical activity is the standard behavioral weight loss approach; however, many fail to achieve clinically meaningful (>5%) weight loss, emphasizing the need for innovative interventions. A recent investigation found that a behavioral weight loss program focusing on 4:3 intermittent fasting (4:3 IMF) produced 60% greater weight loss among healthy adults with OW/OB with no history of cancer than a program focused on daily caloric restriction. Given these promising results and the need for innovative weight loss strategies among breast cancer survivors, studies testing 4:3 IMF among breast cancer survivors are warranted.

**Objective:**

This investigation aimed to evaluate the feasibility, acceptability, and safety of a 4:3 IMF behavioral weight loss program tailored to breast cancer survivors, and to examine exploratory clinical outcomes (eg, body weight, diet adherence, and physical activity) in relation to prespecified thresholds that will inform whether adjustments to the program are needed prior to progression to a larger trial.

**Methods:**

In this proof-of-concept feasibility study, individuals with stage I-III breast cancer who have OW/OB and are post–primary therapy will enroll in a 6-month behavioral weight-loss intervention using a 4:3 IMF dietary strategy. Participants will complete a modified fast of approximately 500 kcal per day on 3 non–consecutive days per week, with ad libitum intake the other 4 days per week. Participants will receive virtual, group-based education from a registered dietitian nutritionist that includes a focus on cancer-specific nutrition guidelines. They will also receive in-person, individualized exercise support from an established exercise oncology program to target cancer-specific exercise guidelines. Intervention feasibility will be assessed via participant recruitment, retention, and education class attendance. Acceptability of Intervention Measure, Intervention Appropriateness Measure, Net Promoter Scores, and focus group interviews will be used to assess program acceptability. Proof-of-concept will be evaluated based on whether prespecified thresholds for changes in exploratory clinical outcomes are met. This information will be used to inform progression to a larger trial.

**Results:**

This study was funded in January 2022. Data collection began in May 2024 and is ongoing as of April 2026. Recruitment was completed in September 2025 with 31 participants. Study volunteers are currently participating in the 6-month intervention, with data collection anticipated to be completed by Summer 2026, data analysis to begin in Summer/Fall 2026, and initial study results expected by early 2027.

**Conclusions:**

This study aimed to examine feasibility, acceptability, and safety of a 4:3 IMF intervention among breast cancer survivors. Data from this study will be used to refine the 4:3 IMF intervention for delivery in a future randomized efficacy trial.

## Introduction

### Background

It is estimated that 70% of breast cancer survivors have overweight or obesity (OW/OB) [1]. At the time of diagnosis, each 5-unit increase in BMI leads to an 8% increased risk of breast cancer recurrence or death [2]. During and after treatment, many breast cancer survivors report weight gain due to treatment-related effects [3-5]. Moreover, breast cancer survivors also face an elevated risk of developing secondary chronic health conditions, including cardiovascular disease and type 2 diabetes [6,7]. This increased chronic disease risk can be explained in part by shared disease risk factors as well as specific effects of breast cancer treatment and is further exacerbated by OW/OB [6,8]. When compared with their normal weight counterparts, breast cancer survivors with OW/OB also report reduced health-related quality of life and physical function, as well as increased fatigue and mood disturbances [9-13]. Due to the rapidly accumulating evidence supporting the negative effects of OW/OB on breast cancer–related outcomes, effective weight management strategies for breast cancer survivors hold substantial promise for enhancing long-term health in this population.

Weight loss of 5%‐10% for breast cancer survivors is recommended to reduce the risk of comorbidities (including cardiovascular disease and type 2 diabetes) and cancer recurrence and improve overall survival, while aligning with current guidelines for obesity treatment [14]. While daily caloric restriction (DCR) plus increased physical activity (PA) remains the standard weight management recommendation for both breast cancer survivors and those with no history of cancer, adherence to DCR + PA–based lifestyle programs declines within 1‐4 months regardless of diet macronutrient content [15]. Moreover, most individuals using DCR will regain a significant amount of weight within 1 year [16-18]. As such, few breast cancer survivors are able to achieve and sustain 5%‐10% weight loss with current lifestyle approaches. Therefore, there is a need for alternative lifestyle programming that can effectively induce weight loss and ameliorate the negative effects of excess body weight on health and quality of life for breast cancer survivors with OW/OB.

Intermittent fasting (IMF) is an alternative dietary weight loss approach that involves cycling between significant (>75%) energy restriction on “fast” days and ad libitum energy intake on “fed” days. IMF differs from time-restricted eating (TRE), a paradigm in which eating is restricted to a window of typically <8‐10 hours each day. Various IMF paradigms have been studied for weight loss efficacy, including 2 nonconsecutive fast days per week (5:2 IMF), 3 nonconsecutive fast days per week (4:3 IMF), and fasting every other day (alternate-day fasting). We recently compared weight loss generated by 4:3 IMF with DCR over 12 months in 165 adults with OW/OB with no history of cancer [19]. The targeted weekly energy deficit was equivalent, and both groups were provided with a guidelines-based comprehensive behavioral weight loss program. Over 12 months, 4:3 IMF exhibited significantly greater weight loss and greater objectively measured percentage caloric restriction (measured via the doubly labeled water intake balance method) compared with DCR, suggesting that the 4:3 IMF paradigm was easier to adhere to over time. Given the success of 4:3 IMF among adults with OW/OB with no history of cancer, we now aim to test the generalizability of these effects among breast cancer survivors with OW/OB in an Obesity-Related Behavioral Intervention Trials (ORBIT) model phase IIa/b proof-of-concept pilot and feasibility study. Therefore, the goal of the present investigation is to evaluate the feasibility, acceptability, and safety of incorporating a 4:3 IMF dietary weight loss intervention with an established exercise oncology program, and to determine whether prespecified progression thresholds based on exploratory clinical outcomes are met to inform whether adjustments to the program are needed prior to progression to a larger trial. While extensive evidence supports the efficacy of our in-house exercise oncology program, *BfitBwell*, to improve physical function and quality of life, research does not support that this program induces weight loss [20,21]. Thus, the integration of a weight loss curriculum with the *BfitBwell* program could provide an effective option to increase PA and induce weight loss in patients with breast cancer. We hypothesize that the intervention will be feasible and acceptable to breast cancer survivors and that 4:3 IMF will meet the prespecified progression thresholds supporting future testing (ie, mean weight loss of ≥5%, achievement of ≥2 modified fast days per week, and increase in moderate to vigorous physical activity [MVPA] of ≥100 minutes per week).

### Study Objectives

The aims of this study are to (1) determine the feasibility, acceptability, and safety of the intervention; (2) evaluate whether a lifestyle weight loss program with 4:3 IMF as the dietary strategy can achieve prespecified progression thresholds (weight loss, adherence to 4:3 IMF, and increases in MVPA) in breast cancer survivors with OW/OB; (3) explore implementation outcomes to inform planning of a future randomized efficacy trial, using dissemination and implementation (D&I) principles; and (4) refine intervention components based on program feedback for delivery in a future randomized efficacy trial. To guide the measurement of D&I principles, we applied the expanded reach, efficacy/effectiveness, adoption, implementation, and maintenance (RE-AIM) framework of implementation outcome dimensions that includes a model of key contextual determinants of successful implementation: the Practical, Robust Implementation and Sustainability Model (PRISM) [22]. PRISM is a widely used framework in intervention research [23,24] and has been applied in cancer survivorship [25].

## Methods

### Study Setting

Participants will be recruited from the region around Denver, Colorado. All participants will be recruited and studied at a single site, the University of Colorado Anschutz.

### Sample Size

We will recruit up to 36 participants over 12‐18 months in 2 cohorts of 15‐18 participants. The primary focus of a phase IIa/b study is to evaluate feasibility and acceptability of the study procedures and to assess whether prespecified progression thresholds for the exploratory clinical outcomes are met, informing progression to a larger trial [26]. A sample of up to 36 participants was chosen as a reasonable target for recruitment over 12‐18 months, given the study team’s experience in recruiting participants with breast cancer for clinical trials. Although not powered for hypothesis testing, this sample size is consistent with recommendations for pilot and feasibility studies and is sufficient to assess whether prespecified progression thresholds are met [27-29]. To account for potential ineligibility or withdrawal, up to 75 intervention participants may be screened for participation to achieve a target sample size (n=36).

### Eligibility Criteria

Adults with OW/OB (aged 18‐65 years, BMI 25‐45 kg/m^2^) and stage I-III breast cancer diagnosed in the past 10 years will be eligible to participate in this study. Participants will be recruited after completion of standard of care definitive surgery, chemotherapy, radiation, immunotherapy, and/or targeted therapy as appropriate prior to the intervention start date. Current or ongoing use of antiendocrine-directed therapy for breast cancer (including ovarian suppression, tamoxifen, aromatase inhibitors, and selective estrogen degraders) is acceptable. Participants must also have an Eastern Cooperative Oncology Group performance status score of 0 or 1 on a 5-point scale, with higher numbers indicating greater disability [30]. While men will not specifically be excluded, given the relatively high incidence of breast cancer in women (100:1), we expect that most, if not all study participants, will be women [31]. Participants with the following conditions will be excluded: recent (past 6 months) acute coronary event, unstable angina, coronary revascularization, stroke, pulmonary embolism, or hospitalization for heart failure or cardiac arrhythmia; serious arrhythmias, including multifocal premature ventricular contractions (PVCs), frequent PVCs (defined as 10 or more per minute), ventricular tachycardia (defined as runs of 3 or more successive PVCs), or sustained atrial tachyarrhythmia; second- or third-degree A-V block, known corrected QT interval greater than 480 milliseconds, or other significant conduction defects; current symptoms suggestive of cardiovascular disease or unstable angina (eg, chest pain, shortness of breath at rest or with mild exertion, lightheadedness, and syncope); uncontrolled hypertension (defined as diastolic blood pressure greater than 100 mm Hg and systolic blood pressure greater than 160 mm Hg); resting heart rate greater than 100 bpm as measured in duplicate on the screening visit after 5 minutes of rest in a seated position; diabetes (history of type 1 or type 2 diabetes or fasting glucose 126 mg/dL or greater, or hemoglobin A_1c_ 6.5% or greater as measured on screening laboratories) unless on metformin or on dipeptidyl peptidase IV inhibitor monotherapy and well controlled with hemoglobin A_1c_ 8% or less; undiagnosed hypo- or hyperthyroidism (thyroid-stimulating hormone outside of the normal range as measured on screening laboratories) or history of uncontrolled thyroid disorder (history of thyroid disease or current thyroid disease treated with a stable medication regimen is acceptable); stage 4 or 5 chronic kidney disease as assessed by estimated glomerular filtration rate less than 30 as measured on screening laboratories; triglycerides greater than 500 mg/dL or low-density lipoprotein cholesterol greater than 200 mg/dL as measured on screening laboratories; clinically significant abnormalities in hematocrit or hemoglobin, white blood cell count, platelets, serum sodium, potassium, or bicarbonate as measured on screening laboratories; presence or history of other metabolic or chronic health problems that would impact ability to safely participate in a weight loss intervention involving an IMF diet and exercise intervention including significant cardiac valvular disease or heart failure and significant gastrointestinal, pulmonary, renal, musculoskeletal, neurologic, hematologic, orthopedic, or psychiatric disease; other active cancer; sustained use of prescription or over-the-counter medications known to *significantly* impact appetite, weight, or energy metabolism (eg, antiobesity medications, appetite suppressants, lithium, antipsychotics, and tricyclic antidepressants) with the exception of antiendocrine–directed treatment for breast cancer; sustained use of systemic glucocorticoids (current or in the past 6 months) unless physiologic replacement therapy for adrenal insufficiency; previous obesity treatment with metabolic bariatric surgery or weight loss device, except (1) liposuction and/or abdominoplasty if performed greater than 1 year before screening, (2) lap banding if the band has been removed greater than 1 year before screening, (3) intragastric balloon if the balloon has been removed greater than 1 year before screening, (4) duodenal-jejunal bypass sleeve, if the sleeve has been removed greater than 1 year before screening, or (5) AspireAssist or other endoscopically placed weight loss device if the device has been removed greater than 1 year before screening; history of clinically diagnosed eating disorders including anorexia nervosa, bulimia, and binge eating disorder (score greater than 20 on the Eating Attitudes Test-26 [32] or pattern of response on the Questionnaire of Eating and Weight Patterns-5 [33] suggestive of possible binge eating disorder or bulimia will require further assessment by the study MD to determine whether it is appropriate for the volunteer to participate in the study); current severe depression or history of severe depression within the previous year, based on the *DSM-IV-TR* (*Diagnostic and Statistical Manual of Mental Disorders, Fourth Edition, Text Revision*) criteria for Major Depressive Episode [34] or history of other significant psychiatric illness (eg, psychosis, schizophrenia, mania, and bipolar disorder), which in the opinion of the study MD would interfere with ability to participate in the diet and exercise interventions; and current alcohol or substance abuse. Women who are pregnant, lactating, or planning pregnancy will also be excluded.

### Recruitment

Participants will be recruited from the University of Colorado Anschutz Cancer Center (CUCC), as well as from the surrounding Denver Metro Area. Flyers with information about the study will be posted in the CUCC Diane O’Connor Thompson Breast Center. The study will also be posted on the CUCC clinical trials website so that it is accessible to patients seeking clinical trials related to breast cancer. We will also provide information about the study to all patients with breast cancer referred to the Anschutz Health and Wellness Center (AHWC) *BfitBwell* Cancer Exercise program as an additional layer of recruitment. We will also recruit from the Metro Denver Area by registering the trial at ClinicalTrials.gov (NCT06399276) and via print and social media advertisements (eg, Facebook, Instagram, etc). The advertisement will include a link to a study landing page providing more information about the study, including a brief description of the study requirements and eligibility criteria. Interested individuals will be provided a link to a screening questionnaire.

### Study Procedures

This 6-month, single-arm trial serves as both a proof-of-concept study and a feasibility pilot study (ORBIT model phase IIa/b) [26] of a lifestyle weight loss intervention using 4:3 IMF as the dietary strategy (ie, modified fast of approximately 500 kcal per day on 3 non–consecutive days per week with ad libitum intake the other 4 days per week). All participants will receive group-based behavioral support weekly for months 0‐3 and biweekly for months 4‐6. All participants will also receive individualized PA support sessions weekly during months 0‐3 and monthly during months 4‐6. This study will not use randomization or blinding as this study is intended to determine only the feasibility, acceptability, safety, and prespecified progression thresholds of the proposed intervention. Detailed descriptions of each study element and assessment measures are provided in the following sections.

### 4:3 IMF Dietary Prescription

Participants will be provided a calorie goal of 500 kcal per day in women and 600 kcal per day in men on 3 nonconsecutive self-selected fast days per week for the duration of the intervention. This goal is designed to produce an approximately 75%‐80% energy deficit from baseline weight maintenance energy requirements on fast days. This will result in an average targeted weekly energy deficit of about 30%, which is consistent with the dietary energy deficit recommended in current obesity treatment guidelines [14]. Participants will be encouraged to consume most of their calories in a single eating occasion (eg, in their dinner meal) on fast days. Sample fast day menus will be provided, and participants will be instructed in calorie counting and will be asked to log their food and beverage intake on fast days only. Participants will consume an ad libitum diet on the other 4 days per week but will be encouraged to make healthy food and portion choices. Suggested macronutrient content will be 55% carbohydrate, 15% protein, and 30% fat.

### PA Prescription

Participants will receive a recommendation to gradually increase moderate intensity aerobic PA to 150‐300 minutes per week. They will also receive a recommendation to complete at least 2 weekly 45- to 55-minute exercise sessions focused on resistance and flexibility training. These aerobic and resistance training targets align with current guidelines for cancer survivors [10,35] as well as current guidelines for weight management [36,37]. Participants will be instructed in how to use a relative intensity scale [36] to achieve the target of moderate intensity. The aerobic exercise target will be individualized based on participants’ baseline level of PA and functional status.

### Time2Bwell 4:3 IMF Survivorship Weight Management Program

All individuals will complete an in-person 90-minute group orientation to introduce the program and review study requirements. To improve adherence to the dietary protocol, individuals will receive a comprehensive group-based behavioral weight loss program that fulfills all current recommendations for behavioral interventions for the treatment of obesity [14]. They will receive 60- to 90-minute education sessions weekly for months 0‐3 and twice per month in months 4‐6. Sessions will be delivered remotely via Zoom (Zoom Communications, Inc) by a registered dietitian nutritionist (RDN) with experience in leading group-based behavioral weight loss interventions. In addition to content on weight loss, sessions provide information on evidence-based lifestyle recommendations for cancer prevention and survivorship put forth by leading organizations (eg, American Cancer Society and American Institute for Cancer Research) [38-40]. The survivorship weight management curriculum was adapted from the publicly available Centers for Disease Control and Prevention 12-month lifestyle weight management program, *PreventT2* [41], to address the needs of cancer survivors with OW/OB. The survivorship weight management curriculum was developed by a PhD nutrition scientist and RDN (EBH) with experience in cancer research using a human-centered design process comprising semistructured interviews and iterative curriculum review and was tested in a 12-week single-arm proof-of-concept trial (*BfedBwell*, NCT06191666). The base curriculum uses moderate DCR as the primary weight loss strategy and promotes weight loss through behavioral modification based on Social Cognitive Theory [42]. Strategies to promote adherence include 26 behavior change techniques, including goal setting, self-monitoring, and environment restructuring [43,44]. Topics covered in the program include realistic weight loss goal setting, portion control, basic nutrition, lifestyle recommendations for survivorship, cognitive restructuring, improving personal food environments, and social networks, and strategies to overcome barriers to healthy eating (Table 1). The *BfedBwell* base curriculum was further refined by the principal investigator (VAC), a PhD nutrition scientist and RDN (EBH) with experience in cancer research, and an RDN with experience teaching 4:3 IMF (KB) to incorporate 4:3 IMF dietary strategies based upon our successful randomized clinical trial in adults with no history of cancer [19,45], including development of 500 kcal meals and strategies to promote satiety to create the *Time2BWell* 4:3 IMF curriculum. Participants will also receive two to three 60- to 90-minute in-person group skills development sessions which will include cooking demonstrations in an instructional kitchen to reinforce content and provide additional behavioral support. Participants will also receive two to three 30-minute one-on-one remote counseling sessions with the RDN during the study period. At each session, the RDN will use a standardized protocol to review individual goals and progress to provide tailored support using motivational interviewing strategies and medical nutrition therapy for weight management as appropriate.

**Table 1.** *Time2Bwell* 4:3 intermittent fasting group–based curriculum.

Program week	Title/**topic**
0	Orientation
1	Getting started with 4:3 IMF^a^
2	Nutrition 101/calorie balance, macronutrients
3	Food cues and portion sizes
4	Spices, flavors, and cultural cuisines
5	Clinical guidelines and cancer prevention/survivorship recommendations
6	Food labels, micronutrients, and meal replacements
7	Moderation and meal planning
8	Mental and emotional health, stress, and mindfulness
9	Foods to include
10	Foods to limit, fad diets, myths, misinformation, dietary supplements, and alcohol
11	Treatment-related and late effects, navigating the transition, and normalization of experiences
12	Budget-friendly meals and recipe development
13	Social support and boundaries
15	Dining out
17	Dietary fats
19	Environment
21	Identity
23	Mindful eating
25	Maintenance of behaviors

a IMF: intermittent fasting.

### *BfitBwell* Cancer Exercise Program PA Support

Individualized support for PA will be provided by the existing clinical AHWC and CUCC *BfitBwell* Cancer Exercise program [20]. Participants will meet individually or in small groups with an exercise oncology specialist from the *BfitBwell* program at the AHWC Fitness Center weekly for months 0‐3 and monthly for months 4‐6. Participants will also receive virtual PA support through the online platform True Coach, designed to complement the in-person sessions. *BfitBwell* is designed in accordance with the American College of Sports Medicine and the National Comprehensive Cancer Network Guidelines for safe exercise in cancer survivors [10,35,46,47]. The resistance and flexibility training exercises and the weekly moderate-intensity aerobic PA targets will be selected after an initial assessment with the *BfitBwell* staff and will be individualized based on baseline level of PA, fitness, functional status, participant preference, and level of adherence during the progression. Exercises and PA targets will be adapted, as necessary, during the intervention based on assessed participant responses.

Behavioral support for PA will also be provided as part of the *Time2Bwell* group-based sessions. Content on health and weight benefits of PA, strategies to overcome barriers to increasing PA, and support for increasing self-efficacy and goal setting for exercise will be discussed. In addition, progress through the *BfitBwell* Cancer Exercise program will also be shared to provide additional support and motivation. Participants will be guided in strategies to adjust exercise intensity and duration on fast days as needed.

### Outcomes

This study is a single-site, single-arm trial designed using the ORBIT model for behavioral treatment development as a phase IIa/b proof-of-concept pilot and feasibility study. Therefore, the primary outcomes are feasibility, acceptability, and safety of the study protocol and to examine prespecified progression thresholds, based on exploratory clinical outcomes to inform whether program modifications are needed prior to progression to a larger trial. The study team operationalized the prespecified progression thresholds as (1) mean weight loss of ≥5%, (2) achievement of ≥2 modified fast days per week, and (3) increase in MVPA of ≥100 minutes per week. These thresholds are not intended to serve as a proxy for estimating preliminary efficacy. Rather, they will be used to inform decisions regarding a future fully powered randomized efficacy trial comparing 4:3 IMF with DCR in breast cancer survivors. These variables will also be examined as key exploratory clinical outcomes. Additional clinical outcomes relevant to the proposed protocol will also be examined (Table 2) [26]. To improve D&I potential, we will apply the expanded RE-AIM framework of implementation outcome dimensions that includes a model of key contextual determinants of successful implementation [22]. Consistent with D&I methodology, we will use multiple methods research [48] to provide insight for refinements. We will also explore implementation outcomes [49] to inform planning of a future trial and iteratively refine intervention components based on program feedback.

**Table 2.** RE-AIM^a^ dimensions and their associated measures.

Justification (RE-AIM dimension) and associated outcome	Measure	Methodology
**Feasibility & acceptability outcomes: Reach**		
Research protocol feasibility	Enrollment	Number of participants screened and proportion of participants that enroll. These data will be tracked by study staff via REDCap^b^.
	Retention	Retention of enrolled participants in the weight loss program and reasons for attrition. These data will be tracked by study staff via REDCap.
	Attendance	Attendance at registered dietician sessions and BfitBwell exercise sessions will be tracked by the study RDN^c^ and BfitBwell program staff.
	Performance of outcome measures	Rates of completion of clinical outcome measures within a 2-week window will be assessed by study staff.
Safety	Patient report	Study-related AEs^d^ will be recorded using case forms by study staff and adjudicated systematically regarding severity (mild, moderate, severe, or serious) and attribution (definitely related, probably related, unlikely to be related, or unrelated to the study interventions) by the study’s principal investigator.
Intervention acceptability	Quantitative program acceptability	Participant satisfaction of (1) content of the 4:3 IMF^e^ dietary support curriculum, (2) content of the BfitBwell program, (3) RDN meeting frequency and structure, and (4) likelihood of continuing the 4:3 IMF and MVPA^f^ prescriptions. Data will be collected via REDCap Survey.
	Qualitative program acceptability	2‐4 x 90 min focus group interviews (6‐8 participants) using content analysis methodology. Topics include experiences/perceptions of the intervention, suggestions for improving the program, and barriers/facilitators of adhering to the intervention. Focus group interviews will be digitally recorded and transcribed verbatim for qualitative analysis by study staff.
**Prespecified progression thresholds: Effectiveness (proof-of-concept)**		
Weight loss	Change in body weight	Measured using digital scale accurate to ±0.1 kg, in the morning, in a fasted state. These data will be collected by study staff.
Dietary adherence	Fast days/week	7-day food records analyzed using Nutrition Data System for Research software by the CCTSI^g^ Nutrition Core. Number of fast days achieved per week (defined as days with energy intake ≤600 kcal in men and ≤500 kcal in women) will be calculated.
Physical activity (MVPA)	Minutes/week	MVPA (min/wk) measured with the activPALv4 activity monitor (PAL Technologies; Glasgow, Scotland) for 7 consecutive days. Data will be analyzed using the associated activPAL software.
**Additional clinical outcomes: Effectiveness (exploratory)**		
Anthropometrics	Waist circumference	Tape measure at the top of the iliac crest. Data will be collected by study staff.
	Fat mass, fat free mass	Dual energy x-ray absorptiometry (DXA, Hologic Horizon W version Apex 5.6.04).
Clinical indicators of health	Blood pressure	Manual sphygmomanometer (average of 2 seated values taken after 5 min rest) will be assessed by study staff.
	Lipid panel, glucose, insulin, HbA_1C_, hs-CRP^h^ HOMA-IR^i^	Morning fasted blood draw analyzed by the Clinical and Translational Research Center or the University of Colorado Hospital Laboratories. HOMA-IR will be calculated as ([insulin] x [fasting glucose x 0.055]/22.5). Data will be collected by a trained phlebotomist.
Dietary intake	Energy intake & macronutrient content	7-day food records analyzed using Nutrition Data System for Research software by the CCTSI Nutrition Core.
	Skin carotenoids	Skin carotenoids will be measured in triplicate using standard methods via a Veggie Meter device.
Functional status	Physical fitness & function	Assessments of cardiorespiratory fitness, and physical functioning. Data will be collected by BfitBwell staff.
Subjective measures	Patient-reported outcomes	Questionnaires related to quality of life, fatigue, mood, stress, sleep, and eating behaviors. Data will be collected via REDCap Survey.

a RE-AIM: reach, efficacy/effectiveness, adoption, implementation, and maintenance.

b REDCap: Research Electronic Data Capture.

c RDN: registered dietitian nutritionist.

d AE: adverse event.

e IMF: intermittent fasting.

f MVPA: moderate to vigorous physical activity.

g CCTSI: Colorado Clinical and Translational Sciences Institute.

h hs-CRP: high-sensitivity C-reactive protein.

i HOMA-IR: homeostatic model assessment of insulin resistance.

#### Primary Outcome: Feasibility and Acceptability

##### Enrollment

The number of participants screened in proportion to the number of participants who enroll will be tracked during and at the end of each recruitment window.

##### Retention

Retention of enrolled participants in the weight loss program and reasons for attrition will be monitored during the 6-month intervention.

##### Adherence and Attendance

Attendance at the RDN-led *Time2Bwell* group sessions and *BfitBwell* exercise sessions will be tracked throughout the entire 6-month intervention.

##### Performance of Outcome Measures

Rates of completion of clinical outcome measures within a 2-week window will be assessed at months 3 and 6.

##### Safety Measures

Study-related adverse events (AEs) will be tracked and adjudicated systematically throughout the 6-month intervention.

##### Quantitative Program Acceptability Measures

Participant satisfaction will be measured on (1) content of the RDN-delivered 4:3 IMF *Time2Bwell* dietary support curriculum, (2) content of the *BfitBwell* Cancer Exercise Program, (3) RDN meeting frequency and structure, and (4) likelihood of continuing the 4:3 IMF and MVPA prescriptions. This measure will be administered via a REDCap (Research Electronic Data Capture) survey emailed to participants weekly during months 0‐3 and following each bimonthly class during months 4‐6. Participants will also complete the Acceptability of Intervention questionnaire [50], Intervention Appropriateness questionnaire [50], and Net Promoter Score questionnaire [51] at months 3 and 6.

##### Qualitative Program Acceptability Measures

Ninety-minute focus group interviews (6‐8 participants per focus group) will be conducted virtually using Zoom videoconferencing. Interview guides will be informed by RE-AIM and PRISM [22]. Topics will include experiences and/or perceptions of the intervention, suggestions for improving the program, and barriers or facilitators of adhering to the intervention, and will primarily target the PRISM dimension of the recipient’s perspective of the intervention (Multimedia Appendix 1). Interviews will be digitally recorded and transcribed verbatim for qualitative analysis.

### Primary Outcome: Prespecified Progression Thresholds

#### Body Weight

Weight (kg) will be measured in clinic in the morning in a fasted state using a digital scale accurate to ±0.1 kg (calibrated yearly) at baseline and months 3 and 6. Mean weight loss of <5% indicates that program modifications are needed prior to future testing.

#### Dietary Adherence

Dietary energy intake will be assessed using a food record for 7 consecutive days at baseline and months 3 and 6 to assess adherence to the 4:3 IMF dietary intervention. Participants will record all food intake on both fed and fasted days. Energy intake will be calculated by the Colorado Clinical and Translational Sciences Institute Nutrition Core using Nutrition Data System for Research software (Nutrition Coordinating Center). Dietary adherence will be assessed as the number of fast days per week (defined as days with energy intake ≤600 kcal for men and ≤500 kcal for women). Achievement of less than 2 modified fast days per week indicates that program modifications are needed prior to future testing.

#### Moderate to Vigorous Physical Activity

PA will be assessed via activPAL v4 accelerometers worn for 7 days prior to each data collection visit at baseline and months 3 and 6. The activPALv4 (PAL Technologies) will be used to estimate time spent in MVPA, light activity, and sedentary behavior. The activPAL is a small (23.5 × 43 × 5 mm) and light (10 grams) device that uses accelerometer-derived information about thigh position to estimate time spent in different body positions (ie, sitting or lying, standing, and stepping). The device is attached to the anterior thigh and is waterproofed by wrapping in a nitrile sleeve. Thus, it can be worn during bathing and overnight, allowing for 24-hour measurement of wake and sleep behavior. The time-stamped “event” data file from the activPAL software will be used to determine time spent sitting, standing, and stepping per day. Using the activPAL program, the event data file will be converted to a second-by-second file, and additional metrics of sedentary behavior (eg, breaks in sedentary time, average duration of sedentary bouts, and sleep time) and time in PA intensity category (sedentary, light, and MVPA) will be estimated. An increase in MVPA of <100 minutes per week indicates that program modifications are needed prior to future testing.

### Additional Exploratory Outcomes

The variables used to assess prespecified progression thresholds (ie, weight, dietary adherence, and PA) are designated as primary outcomes for determining progression to a larger trial but will also be examined descriptively as exploratory clinical outcomes. Additional exploratory outcomes relevant to the proposed trial are described in the following sections.

#### Body Composition

Fat mass and fat-free mass will be measured using dual energy x-ray absorptiometry (Hologic Horizon W version Apex 5.6.04) at baseline and months 3 and 6.

#### Waist Circumference

Waist circumference will be measured with a tape measure at the iliac crest at baseline and months 3 and 6.

#### Laboratory Data

Blood will be collected after a 12-hour fast via venipuncture at baseline and months 3 and 6. Laboratory values assessed will include lipid profiles, glucose, insulin, hemoglobin A_1c_, and high-sensitivity C-reactive protein. A homeostasis model assessment of insulin resistance will be calculated as ([insulin] × [fasting glucose × 0.055]/22.5). Serum, plasma, and whole blood samples will be stored at −80 degrees for future research.

#### Blood Pressure

Blood pressure will be measured using a manual or automatic sphygmomanometer (average of 2 seated values) at baseline and months 3 and 6.

#### Energy Intake and Diet Macronutrient Content

Dietary energy and macronutrient intake will be quantified from 7-day food records collected at baseline and months 3 and 6 and analyzed by the Colorado Clinical and Translational Sciences Institute Nutrition Core using Nutrition Data System for Research software.

#### Skin Carotenoid Score

Estimates of skin carotenoid concentrations will be obtained via reflection spectroscopy in triplicate using standard methods on a calibrated Veggie Meter device (Longevity Link Corporation) [52].

#### Measures of Physical Fitness and Function

These will be collected using a standard physical assessment performed by the *BfitBwell* Cancer Exercise Program. These measures include the 6-minute walk test and the 30-second sit-to-stand test [21]. This assessment will be performed pre- and postprogram during the first exercise session and final exercise session with *BfitBwell* exercise staff. These measures will be collected as potential moderators or confounders of exercise outcomes (eg, the influence of baseline fitness) and will provide information about other benefits of the exercise program.

#### 6-Minute Walk Test

The 6-minute walk test is a common measure of gait and functional capacity with participants walking as far as possible on an oval track (length=160 meters) in 6 minutes. It will be performed according to published guidelines except the test will be performed on an oval track instead of in a hallway, which may result in a slight increase in distance. In brief, participants are instructed to walk (not run) as far as they can in 6 minutes. Rests are allowed, and standard encouragement is provided each minute.

#### 30-Second Sit-to-Stand

The 30-Second Sit-to-Stand test is a functional assessment of lower extremity muscle strength. Participants move from a seated position to a standing position as many times as possible in a 30-second period with no upper extremity assistance. The test is performed once.

#### Patient-Reported Outcomes

To measure patient-reported outcomes, which may be impacted by the 4:3 IMF intervention, several validated questionnaires will be administered to participants. Questionnaires will be measured at baseline and months 3 and 6 and include the following: Binge Eating Scale [53], Functional Assessment of Chronic Illness Therapy—Fatigue Scale [54], Functional Assessment of Cancer Therapy—General [55], Profile of Mood States (40-item version) [56], Pittsburgh Sleep Quality Index [57], and the Three Factor Eating Questionnaire [58]. The estimated time to complete all study questionnaires is ≤75 minutes at each time point to limit participant burden.

### Schedule of Assessments

A schedule of assessments is presented in Table 3. In order to minimize participant burden, the study team will obtain only the minimum required information to adequately address the research questions. Moreover, we will consolidate measures so that all assessments can be completed during the study visit at each assessment time point (see Table 3 for anticipated visit durations). Finally, for all survey assessments, participants will be informed that their responses will be intermittently saved, allowing them to pause the survey and complete it at a later time.

**Table 3.** Schedule of assessments.

Assessments	Pre**s**creening (30‐60 minutes)	Screening (60‐120 minutes)	Baseline (150 minutes)	3 **months** (150 minutes)	6 **months** (150 minutes)
Prescreening questionnaire	✓				
36-hour test fast		✓			
Urine pregnancy test		✓			
Informed consent		✓			
Demographics		✓			
Health history and physical examination^a^		✓			
ECG^b^ and resting heart rate		✓			
Screening blood draw		✓			
Screening questionnaires		✓			
Height		✓			
Weight		✓	✓	✓	✓
Blood pressure		✓	✓	✓	✓
Body composition (DXA^c^)			✓	✓	✓
Waist circumference			✓	✓	✓
Veggie meter scan			✓	✓	✓
7-day food records			✓	✓	✓
7-day activPAL assessment			✓	✓	✓
Outcome blood draw			✓	✓	✓
Outcome questionnaires^d^			✓	✓	✓
Functional assessments			✓	✓	✓
Focus groups				✓	

a Adverse events will be continuously assessed as they are reported to study staff.

b ECG: electrocardiogram.

c DXA: dual energy x-ray absorptiometry.

d Quantitative program acceptability will be assessed weekly during months 0‐3 and following each bimonthly class during months 4‐6. Participants will also complete the Acceptability of Intervention questionnaire [50], Intervention Appropriateness questionnaire [50], and Net Promoter Score [51] at months 3 and 6.

### Ethical Considerations

All protocols and procedures to be used in this study have been approved by the Colorado Multiple Institutional Review Board (protocol no. 23‐1546). All participants will be required to provide their written informed consent before engaging in any study-related procedures. Data that could be used to identify a specific study participant will be held in strict confidence within the research team. No personally identifiable information from the study will be released to any unauthorized parties. With the participant’s approval and as approved by the Colorado Multiple Institutional Review Board, biological samples, clinical and outcome assessment data, deidentified transcripts from audio or visual recordings, and participant health and demographics data and quantitative responses from questionnaires will be stored for at least 3 years after completion of the study. Participants will be compensated for completion of 3- and 6-month outcome measures (US $100 per time point) as well as for their participation in the postprogram focus groups (US $50).

### Data Collection Methods

Assessment of outcome measures will take place during in-person visits to the AHWC clinic and will be performed by trained research staff. To ensure accuracy, anthropometric measures (eg, weight, waist circumference, etc) will be measured twice, and the average of the 2 measures will be calculated for use in analysis. Study questionnaires will be administered via REDCap. Scores for all REDCap questionnaires will be calculated by the study biostatistician. Focus groups will be conducted around month 4 of the intervention to capture participant experiences, with semistructured interview guides centered around the assessment of program feasibility and acceptability. Focus groups will be conducted remotely using Zoom videoconferencing and recorded using the Zoom recording feature. Focus groups will be conducted by a trained moderator (DMO) who will have no role in delivering the intervention and is thus naïve to participant experiences. Audio interview data will be transcribed verbatim by Landmark Associates.

### Statistical Methods

#### Feasibility and Acceptability

##### Enrollment

We will describe enrollment of participants meeting study inclusion and exclusion criteria, including age, sex, race, and ethnicity of participants, over the proposed 12‐18 months time frame. If total enrollment is substantially less than 36 participants, we will determine barriers to enrollment and reevaluate the study design and/or recruitment strategies to enhance recruitment accordingly. We will also assess the participation rate (number approached vs enrolled) and reasons potential participants decline enrollment. Strategies to enhance enrollment may include increasing participant compensation, identifying additional sites from which to recruit participants, and/or budgeting for advertising in the full-scale trial.

##### Retention

We will assess the participant drop-out rate and reasons for attrition over the 6-month intervention. Descriptive statistics will be performed on dropout rate. If more than 20% of participants drop out, we will consider adjusting screening procedures, inclusion and exclusion criteria, or modifying the intervention accordingly to reduce attrition.

##### Attendance

We will use descriptive statistics to evaluate attendance to group videoconference *Time2Bwell* 4:3 IMF behavioral support sessions and *BfitBwell* exercise support sessions; mean attendance <60% will indicate that the program will require major improvements in acceptability prior to the larger study.

##### Performance of Outcome Measures

We will use descriptive statistics to evaluate rates of completion of the outcome measures within a 2-week window at months 3 and 6. If less than 90% of active participants complete outcome measures, study procedures will be adjusted depending on reasons for missing data (eg, implementing additional procedures for scheduling or tracking outcome measures, ensuring a higher study staff-to-participant ratio in the large-scale trial, increasing compensation for completion of outcome measures, or decreasing participant assessment burden).

##### Safety

Overall rates of study-related mild, moderate, severe, and serious AEs will be described. The occurrence of any protocol-related unanticipated AEs or any protocol-related severe or serious AEs will prompt reevaluation of the aspect of the intervention related to the AE.

##### Quantitative and Qualitative Assessment of Program Acceptability

Descriptive statistics will be performed on satisfaction ratings with aspects of the integrated lifestyle weight loss program (including the diet, exercise, and behavioral support components) obtained weekly during months 0‐3 and following each bimonthly class during months 4‐6, as well as the Acceptability of Intervention [50], Intervention Appropriateness [50], and Net Promoter Score questionnaires [50,51]. Qualitative analysis of the focus group transcripts will follow a team-based content analysis approach using ATLAS.ti software (Lumivero, LLC) [59,60]. Coding will involve both a deductive and inductive approach. Deductive codes will be developed a priori to identify quotes around feasibility and acceptability of program components, as well as RE-AIM domains and PRISM determinants. Inductive codes will be developed based on data from the transcripts. The analytical team will iteratively discuss the codebook and achieve consensus for assigning codes to quotes. Each code will have a written definition to ensure consistency in coding. Once all coding is complete, the analytical team will complete a secondary review and go back through all transcripts to ensure that codes were appropriately applied to each quote. Reports of code queries will be pulled, and the analysts will create written summaries of primary outcomes of interest. We will use a convergent mixed methods design [61] to integrate findings from the survey results with qualitative themes from the content analysis to identify intervention areas for refinement. We will look for parallel constructs or themes, identify differences and similarities, and create a joint display to array the integrated results [62,63].

### Prespecified Progression Thresholds

#### Body Weight

We will use linear mixed effects models (LMMs) with unstructured covariance to examine within-participant change in weight from baseline to 3 and 6 months. Weight will be analyzed in log scale in order to estimate percent weight change. The LMM will include time as a fixed effect. Analyses will follow the intention-to-treat principle, and all enrolled participants will be included in the analysis. Weight loss at month 3 of less than 3% or less than 5% would suggest that major or moderate modifications are required before progressing to a larger study, respectively. In addition to its role in assessing progression thresholds, pre- to postchanges in weight will also be examined descriptively to characterize variability and inform the design of a future trial.

#### Dietary Adherence

We will calculate the number of fast days achieved per week (defined as days with energy intake of ≤600 kcal in men and ≤500 kcal in women) assessed with 7-day written food records at months 3 and 6. Achievement of less than 1 or less than 2 fast days per week at month 3 would suggest that major or moderate modifications are required before progressing to a larger study, respectively. Dietary adherence will also be examined descriptively to characterize variability and patterns of adherence.

#### Moderate to Vigorous Physical Activity

We will use the LMM with unstructured covariance to examine the within-participant change in PA (assessed with activPAL) from baseline to 3 and 6 months. Change in MVPA at month 3 of less than 50 or less than 100 minutes per week would suggest that major or moderate modifications are required before progressing to a larger study, respectively. Exploratory analyses of pre- to postchanges and variability in MVPA will also be conducted.

These proposed analyses are not intended for significance testing or to generate causal inferences as this study is not sufficiently powered for such estimates. Rather, the goal of these analyses is to evaluate whether the intervention produces a clinically meaningful signal. This information will be used to determine whether additional testing is warranted and to generate effect size estimates to inform sample size determinations in a future fully powered trial.

### Additional Exploratory Clinical Outcomes

Additional clinical outcomes will be collected to evaluate feasibility of performing these measures as well as to perform preliminary testing of postintervention change and determine the variability for each outcome, including blood pressure, waist circumference, fat mass, fat-free mass, total cholesterol, high-density lipoprotein cholesterol, low-density lipoprotein cholesterol, triglycerides, insulin, glucose, hemoglobin A_1c_, highly sensitive C-reactive protein, insulin sensitivity (homeostasis model assessment of insulin resistance), and daily dietary energy intake and macronutrient content (% fat, carbohydrate, and protein). We will analyze the change in each outcome measure from baseline to 3 and 6 months using LMMs. Analyses will follow the intention-to-treat principle, and all enrolled participants will be included in the analysis. Standard deviation and postintervention change scores will be used in conjunction with existing literature to assist with planning the future randomized trial. Pearson or Spearman correlation, as appropriate, will be used to assess the correlation of change score in peripheral extracellular matrix biomarkers and weight change. Post hoc, potential subgroup effects by breast cancer type (hormone receptor positive, human epidermal growth factor receptor 2 positive, and triple negative) and stage (I-III), age, race, and ethnicity will be explored.

## Results

This research study was funded by the National Cancer Institute in January 2022 as part of a larger trial. Data collection began in May 2024 and, as of April 2026, is ongoing. Recruitment closed in September 2025 with a total of 31 participants. Data collection is anticipated to be completed by Summer 2026, followed by data analysis beginning in Summer/Fall 2026 with a results manuscript submitted for publication in early 2027.

## Discussion

### Expected Contributions

This study will be the first to evaluate a novel fasting paradigm, 4:3 IMF, among breast cancer survivors. While there are published studies and ongoing trials of “intermittent fasting” in breast cancer survivors, a review of a recent meta-analysis in breast cancer survivors [64] and of clinicaltrials.gov reveals that the vast majority of these studies are either (1) TRE interventions focused on limiting daily eating window to less than 8‐10 hours for weight or metabolic benefits or (2) “fasting mimicking diets” comprising brief periods (1‐4 consecutive days) of significant energy restriction designed to enhance effects or improve tolerability of chemotherapy (ie, not designed to induce weight loss). Although TRE has historically received more attention than IMF in cancer research (eg, NOT-CA-24‐073 Factors Impacting How TRE Influences Cancer Related Outcomes), emerging evidence suggests that 4:3 IMF may be more effective for weight loss than TRE, which has been found to produce inferior or at best similar weight loss to DCR, the current standard of care recommendation [65-67].

In a recent study completed by our research team, we compared weight loss generated by 4:3 IMF with DCR over 12 months among 165 adults (mean age 42, SD 9 years; BMI mean 34.1, SD 4.4; 74% female) with OW/OB with no history of cancer [19]. In this study, the targeted weekly dietary energy deficit was matched (34%) and both intervention arms received comprehensive group-based behavioral support. We found that 4:3 IMF produced significantly greater weight loss than DCR at 12 months (−7.7 kg, 95% CI −9.6 to −5.9 kg vs −4.8 kg, 95% CI −6.8 to −2.8 kg; *P*=.04). Over 12 months, 4:3 IMF exhibited greater objectively measured % caloric restriction than DCR confirming greater dietary adherence. The 4:3 IMF intervention was well tolerated (<5% diet-related AEs, all mild), attrition was lower in 4:3 IMF versus DCR (19% vs 30%), and both groups increased PA and diet quality similarly. The 4:3 IMF group also displayed greater improvements in cardiometabolic health including greater increases in homeostatic model of insulin resistance (mean difference −1.1, SE 0.5 mg/dL). Thus, 4:3 IMF is a well-tolerated alternative dietary strategy that produces superior dietary adherence and weight loss over 1 year compared with DCR in adults with OW/OB with no history of cancer. In this study, we propose to take the first steps toward understanding the generalizability of these findings to other populations by examining the feasibility, acceptability, and safety of 4:3 IMF among breast cancer survivors with OW/OB and determining whether prespecified progression thresholds based on exploratory clinical outcomes are met. Translating these results to breast cancer survivors could expand the range of evidence-based dietary weight loss strategies available to this population, thus addressing a significant research gap.

### Strengths and Potential Difficulties

A strength of this research is the provision of the 4:3 IMF dietary strategy within the context of a behavioral weight loss program consistent with current guidelines for obesity treatment that was modified to include cancer survivorship nutrition and PA guidance. The behavioral weight loss program uses a standardized written curriculum that is scalable and can be delivered remotely by off-site lifestyle weight loss group facilitators trained in the curriculum. Support for increasing PA will be provided by an established exercise oncology program (ie, *BfitBwell*), which does not require specialized equipment and can be adapted to any exercise facility or delivered remotely. Finally, our multiple methods approach will enable us to gain in-depth insights regarding program perceptions from our target population. This information will be used to inform program modifications, ensuring that our program is responsive to the needs and preferences of our intended users before progressing to more costly efficacy testing.

Although this study has several strengths, it is not without limitations. Given the nature of the 4:3 IMF protocol, a relevant concern is our ability to retain breast cancer survivors for the duration of the intervention. In our experience, the most important factor for ensuring adherence and retention in interventional weight loss trials is clearly outlining expectations during the screening process, maintaining excellent communication with participants, and frequent and high-quality interaction between participants and key study staff. If retention is lower than expected, we will consider the following strategies: provision of additional behavioral support, increasing compensation for outcome measures, or other strategies suggested based on exit interviews with participants who drop from the study. Additionally, we recognize that adherence to 4:3 IMF will not be perfect, and we do not expect it to be, as adherence to DCR is typically imperfect as well. The research question we seek to address is not whether breast cancer survivors will fully adhere to 4:3 IMF but rather whether 4:3 IMF is a feasible, acceptable, and safe alternative dietary approach to DCR and to inform future research efforts.

### Future Directions

This study will provide preliminary data to aid further research efforts in (1) adapting this novel 4:3 IMF intervention to the unique characteristics of breast cancer survivors, and (2) informing the development of randomized trials comparing longer-term efficacy of 4:3 IMF with the current standard of care (DCR) in breast cancer survivors with OW/OB. This line of research could culminate in a fully powered, long-term, multicenter trial to evaluate the impact of the 4:3 IMF dietary weight loss intervention on weight and breast cancer–related outcomes among a larger and more diverse sample of survivors.

### Conclusions

More than 70% of breast cancer survivors have OW/OB, which is associated with greater risk for cancer recurrence and death compared with those of normal weight [1,2]. Current weight loss strategies have been minimally effective, necessitating the development of innovative weight loss solutions for this population. This proof-of-concept feasibility study will be the first to assess a novel weight loss paradigm, 4:3 IMF, among breast cancer survivors. Results from this study may be used to broaden the range of evidence-based dietary weight loss strategies available to breast cancer survivors, thus addressing a critical need.

## Supplementary material

10.2196/86281 Multimedia Appendix 1 Postprogram focus group guide.

10.2196/86281 Peer Review Report 1 Peer review report by CPSS - Cancer Prevention Study Section (National Institutes of Health, USA).

## Abbreviations

AE: adverse event
AHWC: Anschutz Health and Wellness Center
CUCC: University of Colorado Cancer Center
D&I: dissemination and implementation
DCR: daily caloric restriction
*DSM-IV-TR*: *Diagnostic and Statistical Manual of Mental Disorders*, *Fourth Edition, Text Revision*
IMF: intermittent fasting
LMM: linear mixed effects model
MVPA: moderate to vigorous physical activity
ORBIT: Obesity-Related Behavioral Intervention Trials
OW/OB: overweight or obesity
PA: physical activity
PRISM: Practical, Robust Implementation and Sustainability Model
PVC: premature ventricular contraction
RDN: registered dietitian nutritionist
RE-AIM: reach, efficacy/effectiveness, adoption, implementation, and maintenance
REDCap: Research Electronic Data Capture
TRE: time-restricted eating
