# Supplementary material for: Exploring the Feasibility, Acceptability, and Safety of a 4:3 Intermittent Fasting Weight Loss Program Among Stage I-III Breast Cancer Survivors With Overweight or Obesity: Protocol for a 6-Month Proof-of-Concept Feasibility Trial
Source: JMIR Res Protoc. 2026 Jul 23;15:e86281. doi: 10.2196/86281 (PMC13395427; doi:10.2196/86281)
Supplement: Multimedia Appendix 1 [file resprot-v15-e86281-s001.docx]

**Multimedia Appendix 1**

***Time2BWell* Program, 3-month Pilot Trial
Participant Focus Group Guide**

**The below script is an overview of the content that we plan to cover in this focus group interview, however, the exact verbiage of the questions and prompts may change when conducting the focus group interviews.

**Focus Groups Questions** ***[Start Recording]***

- State “*Time2BWell* 3-month Pilot trial Conversation Group”, the date and location of focus group.
- Let’s start by going around the room and introducing ourselves. Please share with the group:
  - 1. Your first name
    2. One of your favorite hobbies

**Thank you all for being here today! Remember, for today/tonight’s discussion, we want you to focus on your overall experience in the *Time2BWell* program.**

Thank you all for being here. We are happy to have you! I’m going to start off by providing a brief overview of the Time2BWell program you received and then we will dive into some specific questions about the program.

This program started off with an in-person orientation session. After the in-person orientation, you received weekly group education classes with your group instructor (registered dietitian), delivered via Zoom. The group classes discussed topics such as survivorship nutrition education, adopting a 4:3 intermittent fasting diet, and strategies and skills for diet and exercise behavior change. As part of the intervention, you followed a 4:3 intermittent fasting diet, where you limited your caloric intake to ~500 calories a day for women and 600 calories a day for men on 3 non-consecutive days a week. On the other 4 days per week, you were asked to eat healthy foods and portions but did not have to restrict calories. The *Time2BWell* program also involved two in-person cooking demonstrations, and two individual support sessions with an RD.

In addition, the program included a goal to increase your physical activity level to 150-300 minutes per week of moderate-intensity exercise, a membership to the Anschutz Health and Wellness Center fitness center, and exercise support from an **exercise** program for cancer survivors, called BFitBWell. During the first 3 months of the program, you received weekly in-person supervised exercise sessions and virtual TrueCoach exercise sessions 3-5 times a week as part of BFitBWell exercise support.

Currently, you all are receiving ongoing support in the form of bimonthly group-based weight maintenance support sessions, as well as monthly in-person supervised exercise sessions and virtual TrueCoach exercise sessions 2-3 times a week. You will continue to receive this support until month 6 of the intervention.

I would like to emphasize that my goal today is to listen and to learn from you all. I consider each of you the expert on your lived experience and I want to know more about your experience as a participant in the *Time2BWell* program. There are no right or wrong answers to these questions and please be as honest as possible and your responses will be kept anonymous. We want to hear the good, the bad, and the ugly because that is what will help us make this program the best that it can be. Our team will not be offended if you have anything negative to share and we want to encourage each of you to be totally honest with us about your experience.

HIT RECORD BUTTON

We would like to start off by discussing your thoughts about your experience during the 3-month *Time2BWell* program.

1. What was your **overall experience** like with the *Time2BWell* program? [PRISM –Participant Perspective of the Intervention also Implementation and Sustainability]
   1. What are your thoughts on the **group education classes**? What went well? What didn’t go so well?
      1. We kicked off with an in-person orientation session – what did you all think of that initial in-person session?
      2. How was the duration of the initial weight loss phase of the program (14 weeks) and frequency of classes (weekly) for you?
      3. How was the delivery format (Zoom) for you? What about the time of day it was offered?
      4. How do you feel about the curriculum you received in these classes?
      5. Any specific feedback on the handouts/visuals you received?
      6. Did you feel like you could see yourself in the class materials/handouts? Are there ways we could improve this? [Program fit]
      7. How did you feel about the class instructor? Your responses will be kept anonymous
         1. What are some things your instructor did well?
         2. What are some things your instructor could have done better?
      8. How are the **bimonthly weight maintenance** support sessions going for you?
         1. What other things could we provide to help support you during this weight maintenance phase?
         2. How is the frequency of these maintenance support sessions for you?
         3. Currently you are receiving the bimonthly support sessions for 3 months. In an ideal world, how long would you like to receive this support for?
   2. What are your thoughts on the **2,** **individualized support sessions** you received from the RD? What went well? What didn’t go well?
      1. How was the delivery format (Zoom) for you?
      2. How did scheduling these sessions go for you? Are there ways we could make scheduling easier?

What is the ideal amount of support sessions you would have received?

- 1. What are your thoughts on the **2, in-person** **cooking demonstrations** you received? What went well? What didn’t go well?
     1. How was the delivery format (in-person) for you? What about the time of day it was offered?
     2. To be able to recruit and include more people, we are considering delivering the full program virtually in our next grant. For virtual delivery – we could send a grocery list or do grocery delivery from Safeway or King Soopers so you could cook alongside the demo. What are your thoughts on the virtual delivery of the cooking demos?
     3. What did you think about the recipes and/or skills that were demonstrated?

What is the ideal amount of cooking demonstrations you would have received?

- 1. How much did the Anschutz Health and Wellness center fitness membership entice you to join the study?
     1. How did the membership influence your motivation to stay in the study?
     2. How much did you utilize the fitness center membership outside of the BFitBWell sessions?
  2. What are your thoughts on the **exercise support you received from the BFitBwell staff?** What went well? What didn’t go well?
     1. What did you think about the in-person, supervised sessions?
        1. How did scheduling the in-person sessions go for you? Are there ways we could make scheduling easier?
        2. What are your thoughts on receiving these sessions virtually?
     2. What did you think about the virtual TrueCoach sessions?
        1. What went well? What didn’t go so well?

1. How was your experience completing the **BFitBWell exercise support alongside the** **Time2BWell nutrition support** program? How did this go from your perspective?
   1. What went well about integrating these programs? What didn’t go well?
   2. How did you feel about the balance/focus on diet vs. physical activity? Too much diet? Too much physical activity? Just right?
2. What was your overall experience like following **4:3 intermittent fasting**? How did fasting (limiting caloric intake to 500 calories a day) 3 days a week go for you?
   1. During these past few months, what were some things that made it **harder** for you to adhere to 3 fast days a week?
      1. PROBE for deeper thoughts – What about ____x____ made it harder for you? Tell me more about ___x___.
         1. In what ways could the *Time2BWell* program better support you through these barriers?
      2. PROBE – what other program features might have been helpful? (higher frequency of classes/meetings, an app? Etc.)
   2. Now that we have talked about the things that make it harder to adhere to the 4:3 intermittent fasting, what were some of the things that made it **easier** to adhere to 3 fast days a week over these past few months?
      1. PROBE - In what ways did the *Time2BWell* program support you in your adherence to 4:3 IMF?
      2. PROBE – What others types of support might have been helpful for adhering to 3 fast days a week?
   3. In this protocol, we set the fast day calorie goal at 500 calories because we didn’t measure your resting metabolic rate. Another strategy is to provide a personalized fast day prescription based on resting metabolic rate – this could mean a lower fast day calorie goal for some people (<500 calories a day). Would a lower fast day calorie goal have been feasible?
3. What do you think about the likelihood of you **keeping up with 4:3 intermittent fasting and diet recommendations long-term** (like for the next year)? [RE-AIM, Participant Maintenance; PRISM – Implementation and Sustainability Infrastructure]
   1. PROBE – About how many days a week of fasting do you think you will be able to keep up with long-term? What would the fast day calorie goal be?
   2. PROBE - regarding what might get in the way long-term.
   3. PROBE - regarding what might help long-term.
4. What do you think about the likelihood of you **keeping up with the physical activity recommendations long-term** (like for the next year)? [RE-AIM, Participant Maintenance; PRISM – Implementation and Sustainability Infrastructure]
   1. PROBE - About how many minutes/week of physical activity do you think you will keep up with long-term? Why?
   2. PROBE – About how much strength vs cardio will you plan to do?
   3. PROBE - regarding what might get in the way long-term.
   4. PROBE - regarding what might help long-term.
      1. PROBE – what do you think would best support you in the maintenance phase? Continued access to TrueCoach? Continued access to the fitness center? Continued monthly 1:1 support sessions?
5. What were some of **most important** things you learned in the *Time2BWell* program? [PRISM – Program value – may touch on Participant perspectives on the intervention]
   1. PROBE - Any other important key lessons learned? What else stood out to you from this program?
6. Please tell me, from your perspective, how has the *Time2BWell* program **impacted your health** **and wellbeing**? [PRISM – Program value – may touch on Participant perspectives on the intervention]
   1. PROBE for changes in eating patterns, weight, physical activity, quality of life, stress/mood, physical function and physical fitness, sleep, cancer recurrence, etc.
   2. PROBE – how did your participation in this program impact relationships in your life (with partner, family, friends, co-workers)?
   3. PROBE – How might other breast cancer survivors be impacted if they were to participate in the *Time2BWell* program?
   4. PROBE - Would you recommend the *Time2BWell* program to other breast cancer survivors? Why/why not?
7. This program was provided at no **cost** to you. In the future, we are considering ways to make this program sustainable and to do that, we will need to cover the cost of the staff time to deliver the program and fitness center memberships. If you were asked to pay to participate in the *Time2BWell* program, how much do you think you would have been comfortable paying? What could be a reasonable amount to ask? [PRISM – Implementation and Sustainability Infrastructure]
   1. PROBE – how much would be too much to pay for a program like *Time2BWell*?
8. We are exploring ways to recruit for a larger *Time2BWell* research study in the future. What would be some ways we could effectively **reach** people so they can hear about the research study? [RE-AIM – Reach]
   1. PROBE—How did you hear about the study and what motivated you to pursue the study? (ex: weight loss, eating healthier, increasing exercise, social connection, increased energy, etc.)
   2. PROBE – radio ads, Social Media ads, local Recreational Centers, libraries, churches, any cancer support groups
9. Think back to the beginning of your participation with the *Time2BWell* study, which was ~4 months ago. What did you consider when you agreed to participate in the *Time2BWell* program? [PRISM – Organizational Characteristics of Participants, Participant Perspective of the Intervention, External Environment; RE-AIM – Reach for Participants]
   1. PROBE - Was there anything that you were concerned about by agreeing to participate in the *Time2BWell* study?
   2. PROBE - Was there anything that you were excited about by agreeing to participate in the *Time2BWell* study?
10. This next question is about things in your **environment** that surround you. So, you can think about things like your access to safe places to be active, access to grocery stores, your income, and others. How do you think your environment played a role in your ability to adhere to the *Time2BWell* program? [PRISM – External Environment]
    1. PROBE – What aspects of your environment made it harder (or easier) for you to adhere to the *Time2BWell* program? [intervention fit with environment]
       1. PROBE for physical environment (access to places to be active, access to grocery stores, safety) and social environment (income/financial factors, access to Wi-Fi)
    2. PROBE – What are some resources outside of the *Time2BWell* program that may help you to make changes to your diet and exercise? [PRISM – External Resources]
       1. PROBE for physical environment (access to places to be active, access to healthy food selections, a good pair of shoes) and social environment (income/financial factors, access to Wi-Fi)
11. If you could wave a magic wand, what kinds of changes would you make to the *Time2BWell* program? [PRISM –Participant Perspective on intervention]
    1. PROBE – What kinds of things would you like to offer to other breast cancer survivors who want to lose weight?

**Thank you for that information!** It is very helpful and interesting.

Next

1. I am almost done with all my questions for today/tonight. As a result of our discussions, do you have additional thoughts about your experience with the *Time2BWell* program?
   1. PROBE for details
2. Okay, great. Now, after everything we have talked about today, is there anything we have missed? Is there anything else about this topic that you think we should know?

**Thank you so much** for meeting and for sharing your ideas with us! If there’s anything further that you’d like to add, please let us know by writing your question or comment in the chat and put your name and contact information in the chat if you would like a call back.

Please check your emails in the next few days. We will be sending you a $50 gift card from TangoCard. Thank you again.

*Gift cards will be sent electronically via TangoCard*
