# Supplementary material for: Exploring the Feasibility, Acceptability, and Safety of a 4:3 Intermittent Fasting Weight Loss Program Among Stage I-III Breast Cancer Survivors With Overweight or Obesity: Protocol for a 6-Month Proof-of-Concept Feasibility Trial
Source: JMIR Res Protoc. 2026 Jul 23;15:e86281. doi: 10.2196/86281 (PMC13395427; doi:10.2196/86281)
Supplement: Peer Review Report 1 [file resprot-v15-e86281-s002.pdf]

SUMMARY STATEMENT

PROGRAM CONTACT: **(Privileged Communication)** ***Release Date:*** **07/16/2021**

Revised Date:

[redacted]@mail.nih.gov

Application Number: **1 R01 CA258766-01A1**

Principal Investigators (Listed Alphabetically): ***Formerly:*** **1R01CA258766-01**

CATENACCI, VICTORIA A
KABOS, PETER
MACLEAN, PAUL S. (Contact)

Applicant Organization: UNIVERSITY OF COLORADO DENVER

Review Group: **CPSS**
Cancer Prevention Study Section

Meeting Date: **06/17/2021** ***RFA/PA:*** **PAR21-035**
Council: **OCT 2021** ***PCC:*** **4NNC**
Requested Start: **09/01/2021**

Project Title: **Novel dietary interventions for reducing obesity-associated breast cancer**

SRG Action: **Impact Score:20 Percentile:3**
Next Steps: **Visit https://grants.nih.gov/grants/next_steps.htm**
Human Subjects: **30-Human subjects involved - Certified, no SRG concerns**
Animal Subjects: **30-Vertebrate animals involved - no SRG concerns noted**
Gender: **2A-Only women, scientifically acceptable**
Minority: **1A-Minorities and non-minorities, scientifically acceptable**
Age: **3A-No children included, scientifically acceptable**

Project Year	Direct Costs Requested	Estimated Total Cost
1	[redacted]	[redacted]
2		
3		
4		
5		
TOTAL	[redacted]	[redacted]

ADMINISTRATIVE BUDGET NOTE: The budget shown is the requested budget and has not been adjusted to reflect any recommendations made by reviewers. If an award is planned, the costs will be calculated by Institute grants management staff based on the recommendations outlined below in the COMMITTEE BUDGET RECOMMENDATIONS section.

1R01CA258766-01A1 MACLEAN, PAUL

RESUME AND SUMMARY OF DISCUSSION: This application is addressing an important area of research dealing with the development of novel dietary interventions for reducing obesity-associated breast cancer. Specifically, the multiple investigator (MPI) team proposes to conduct [redacted] and a clinical trial to explore a novel weight loss strategy – intermittent energy restriction (IER) – to [redacted]. The reviewers agreed that the study was supported by rigorous published and preliminary data, and significance was high. If successful, the results could be valuable for improving breast cancer outcomes and could change clinical practice by validating the efficacy and feasibility of the IER approach to weight loss and reduction of obesity-associated breast cancer. The MPI team is highly experienced with complementary expertise in medical oncology, breast cancer, obesity, nutrition, and weight loss interventions. The team was further bolstered by the recruitment of statisticians. The research environment was exceptionally conducive to the proposed work. [redacted]. Methodological innovation was found in the use of [redacted], head-to-head comparison of TRF and IF, [redacted]. The experimental design was rigorous with well-crafted [redacted] and clinical work. The feasibility trial had a well-detailed timeline, with accounted enrollment shortfalls scaled to diverse populations of different racial/ethnic backgrounds and larger clinical/multicenter settings. The specific Aims were well-integrated and independent; [redacted] Aim 1 will inform later aims, and the timeline gives an opportunity to modify Aim 3. [redacted] and Aim 3 has access to a large breast cancer patient population, which was an important asset. Scientific rigor was strong with clearly justified sample sizes adequately powered for clinically relevant improvement (change in body weight). Consideration of sex as a biological variable was appropriate. The panel appreciated the responsiveness to the prior criticisms, especially re statistical power and computational expertise; the application has improved and represents a formal proof-of-concept study design with detailed clinical and [redacted] outcomes. The lack of details on how [redacted] will be defined was a minor weakness that did not detract from enthusiasm for the high significance, clinical relevance, and other strengths of this outstanding application. Following the discussion, the reviewers unanimously agreed that the overall impact of the proposed work on the field of breast cancer prevention is likely to be very high and long-lasting.

[redacted]
